# A Comparison of the Effects of Prolonged Infusion of Propofol 2% and 1% During Total Intravenous Anaesthesia Using Target-Controlled Infusion Technique for Elective Neurosurgery

**DOI:** 10.21315/mjms2022.29.4.8

**Published:** 2022-08-29

**Authors:** Kar Yee Loo, Sanihah Che Omar, Rhendra Hardy Mohamad Zaini, Wan Mohd Nazaruddin Wan Hassan, Praveena Seevaunnamtum

**Affiliations:** 1 Department of Anesthesiology and Intensive Care, School of Medical Sciences, Universiti Sains Malaysia, Kelantan, Malaysia; 2 Hospital Universiti Sains Malaysia, Kelantan, Malaysia

**Keywords:** target-controlled infusion, propofol, neurosurgery, craniotomy, hypertriglyceridemia

## Abstract

**Background:**

Total intravenous anaesthesia (TIVA) with a combination of target-controlled infusions (TCIs) of propofol and remifentanil has been advocated for a favourable neurosurgical outcome. Neurosurgical procedures often involve a prolonged duration and large cumulative infusion of propofol. This study compares the serial serum lipid profile, acid-base balance and lactate level of neurosurgical patients anaesthetised with TCIs of propofol at 2% versus 1%.

**Methods:**

A total of 74 patients who underwent an elective craniotomy under general anaesthesia were randomised into two groups: i) propofol 1% (*n* = 37) and ii) propofol 2% (*n* = 37). All patients were anaesthetised using TCIs of propofol and remifentanil. Serial lipid profiles (serum triglyceride [TG] and cholesterol levels) were taken at the baseline, upon cessation of propofol and at 2 h post-operation. The total dosage, volume used and syringe changes of both groups were also documented.

**Results:**

The total volume of propofol used was significantly lower in the 2% group than the 1% group (157.19 mL [SD = 77.14] versus 335.17 mL [SD = 174.27]; *P* = 0.005) and the frequency of syringe changes was also less in the 2% than the 1% group (2 [3] versus 6 [3]; *P* < 0.001). However, there were no significant differences between the two groups in terms of serial serum TG, cholesterol, the acid-base balance or the lactate level. There was also no significant correlation of lipid profile with cumulative dose or volume of propofol infused between the two groups.

**Conclusion:**

Both concentrations of propofol, 1% and 2%, were comparable in terms of the serial lipid profile, acid-base balance and lactate level during TIVA using TCIs for elective neurosurgery. The benefits of propofol at 2% were that a lower volume was used and there were fewer syringe changes, which could minimise anaesthesia interruption throughout surgery.

## Introduction

Propofol has a favourable pharmacodynamic profile for neuroanaesthesia. It provides a dose-dependent reduction of the cerebral metabolic rate of oxygen (CMRO_2_) and cerebral blood flow and hence reduces the intracranial pressure (ICP) while maintaining flow-metabolism coupling (1, 2). Total intravenous anaesthesia (TIVA) using propofol during neurosurgical procedures facilitates avoidance of intracranial hypertension, preservation of cerebral perfusion pressure (CPP) and provision of the optimal surgical condition, all of which are necessary to prevent further deterioration of the pre-existing neurological issue (3–5). As neurosurgery is aimed at preserving or restoring brain function, the anaesthesiologist is compelled to adopt an anaesthetic agent such as propofol that provides swift induction and smooth, rapid emergence (3, 6).

Fast distribution from the plasma to tissues, rapid redistribution from slow compartments and high metabolic clearance of propofol contribute to the advantages it has of smooth induction and rapid emergence (6). Moreover, owing to its relatively short (though context-sensitive) half-time and short effect-site equilibration time, propofol infusions are readily titratable (6–7). Propofol has come to be widely used in TIVA due to its favourable pharmacokinetic profile. In the context of evolving fields of pharmacotherapy and improving techniques to administer safer anaesthesia for neurosurgical procedures, TIVA that combines target-controlled infusions (TCIs) of propofol and remifentanil has become popular.

Propofol has high lipid solubility with an octanol-water partition coefficient of 6761:1. Due to its poor water solubility, it is formulated in an oil-in-water emulsion, in which the fat emulsion contains long- and medium-chain triglycerides (LCTs/MCTs) at a 1:1 ratio (8–9). Neurosurgical procedures, especially intracranial surgery, often involve a prolonged duration of operation, with a reported mean surgical duration of 3 h 40 min (10). Thus, neurosurgical patients potentially have a higher peri-operative lipid load in comparison to other patients. The potential adverse events of propofol-related hypertriglyceridemia (hyperTG) are pancreatitis (11–14), stroke (15), metabolic acidosis and increased risk of propofol-related infusion syndrome (PRIS), each of which are rare but can be fatal (16–18).

To cut down on lipid loading, propofol at 2% has been introduced. Several clinical studies (19–22) comparing a 2% propofol formulation with the standard propofol at 1% have shown no significant difference in pharmacokinetic parameters. When we specifically consider TIVA for a craniotomy, three comparison studies are available. The first study, contributed by Dewandre et al. (19), compared the effect of 2% and 1% formulations of propofol in TIVA for a craniotomy, using a bolus, elimination and transfer (BET) regime. The study reported that the pharmacological profiles were identical, except for a significantly higher plasma TG concentration found for the 1% propofol group. The serum TG from both groups, nevertheless, remained within the normal range. The second study, by Zattoni et al. (23), also compared the effects of 2% and 1% formulations of propofol on the induction and maintenance of anaesthesia in an elective craniotomy. This study showed that both formulations were similar in terms of the overall administration rates, recovery times, haemodynamic variables and tolerability. The plasma TG levels were reportedly lower in the 2% propofol group compared with the 1% group, and propofol at 2% was proposed to be an acceptable alternative to propofol at 1% in patients undergoing elective craniotomy neurosurgery. The third study, by Sato et al. (24), compared the effects of 1% and 2% propofol in an LCT/MCT emulsion on serum lipids, and reported that the 2% formulation of propofol in the LCT/MCT emulsion was beneficial for reducing the load on lipid metabolism. There is no robust evidence available at the time of writing that compares the two propofol concentrations using the TCIs technique on lipid loading; however, there are studies available comparing different concentrations for conventional TIVA.

This research compared the prevalence of hyperTG among patients using propofol at 2% and 1% for TCIs in elective neurosurgery. We explored the relationship between the serum TG, cumulative propofol dose, volume administered and duration of therapy. The association of the acid-base status with lipid loading from propofol at 1% and 2% was also reviewed.

## Methods

This double-blinded, randomised controlled trial was conducted in the neurosurgical operation theatre of Hospital Universiti Sains Malaysia, Kelantan from December 2019 to October 2020.

We recruited patients listed for elective neurosurgery, with ages ranging between 16 years old and 80 years old, who fulfilled the American Society of Anaesthesiologists’ (ASA) physical classification system at Class 1 or Class 2 and had given consent either themselves or through their legal guardians. The exclusion criteria were as follows: any patient with a known disorder of their lipid metabolism, such as familial hyperTG, a metabolic syndrome, liver disease of an infectious or non-infectious cause, significant cardiac dysfunction, known allergy to propofol, history of alcohol or drug abuse, obstetric patients and the morbidly obese.

The recruited participants were randomised into the propofol 1% and 2% groups, which were revealed on the day of surgery using computer-generated randomisation. The participants were not informed of the group they would be allocated in advance. Due to logistics surrounding the available infusion pump, knowledge of the propofol concentration was relevant to the anaesthetist in-charge. Hence, the allocated group was made known to the anaesthetist in-charge. Nonetheless, the participants, investigator and laboratory personnel running the lipid profile were blinded.

Anaesthetisation of the recruited patients was carried out by anaesthesiology trainees who had at least 3 years of experience of anaesthesia, with guidance from the anaesthetist in-charge. Before induction, an 18G intravenous (IV) cannula was inserted and non-invasive monitoring devices, such as an electrocardiogram, pulse oximetry (SpO_2_), capnography and non-invasive blood pressure measure, were put in place. Propofol (fresofol 1% or fresofol 2%; Fresenius Kabi) was streamlined to the Schnider model with effect-site targeting and co-administrated with remifentanil (Ultiva^TM^; GlaxoSmithKline) for TIVA. The infusion system was a Fresenius Kabi infusion pump (Injectomat^®^ TIVA Agilia). After 3 min of pre-oxygenation with an inspired fraction of oxygen of 100%, total intravenous induction was performed for all patients using a propofol infusion (4 μg/mL and titrate upwards till loss of consciousness), remifentanil infusion (2 ng/mL up to 4 ng/mL) and a single dose of rocuronium (0.5 mg/kg) to facilitate tracheal intubation. During the maintenance phase, mechanical ventilation was adjusted according to the patient’s age and to maintain an end-tidal expired fraction of CO_2_ between 30 mmHg and 35 mmHg, in a gas mixture of 50% O_2_–50% air.

Changes to the propofol and remifentanil infusion rate were left at the discretion of the anaesthetist in-charge, according to the usual clinical signs (change in heart rate and/or blood pressure, movement). The anaesthetist could opt for an incremented or decremented infusion rate from 2.5 μg/mL–6 μg/mL for propofol and 1 ng/mL–8 ng/mL for remifentanil. The propofol and remifentanil doses for induction and maintenance in this study were slightly different compared to those used in a study by Lamperti and Ashiq (25). For propofol, they used 0.5 μg/mL–14 μg/mL and titrated upwards till loss of consciousness for induction and used 3 μg/mL–4 μg/mL up to 7 μg/mL if required for maintenance. For remifentanil, they used 4 ng/mL–7 ng/mL for induction and then 4 ng/mL–7 ng/mL (or up to 10 ng/mL–15 ng/mL in certain cases) for maintenance. An advanced depth of anaesthesia monitoring such as the bispectral index state (BIS) or response entropy was not compulsory and the sensor strip can be difficult to apply in certain neurosurgical cases. Sury (26) recommends using a specific form of anaesthesia monitoring for patients with a neuromuscular blockade, such as an electroencephalogram or the isolated forearm technique, to reduce their risk of awareness during general anaesthesia. In this research, in addition to the standard monitoring, an arterial line was inserted for beat-to-beat monitoring of the blood pressure and a central venous catheter was inserted for administration of multiple drugs depending on the case. Sterofundin was used as the main option for fluid maintenance unless deemed inappropriate by the anaesthetist in-charge, such as in cases of massive blood loss or haemodynamic instability. Instead, in these cases, the anaesthetist matched their choice of crystalloid to eliminate confounding factors.

Blood sampling was done for both groups peri-operatively. For each patient, 2 mL of blood was taken for lipid profiling before induction, upon cessation of the TCIs of propofol and 2 h post-cessation of the TCIs. Additionally, a 2-hourly, 0.2 mL arterial blood sampling was done throughout the period of anaesthesia as per routine. Samples taken were labelled with ‘pre-op’, ‘intra-op’ and ‘post-op’ to indicate the sampling order. All blood samples taken were sent to the chemistry pathology lab for centrifugation within 2 h of sampling to avoid possible cell lysis. The blood samples were analysed using the reagent Magnesium XL, SYS 1 manufactured by Biorex (Mannheim, Germany) and the analyser Architect C8000 by Abbott (Abbott Park, Illinois, U.S.A). Intra-operative events such as hypotension, hypothermia and hypoxia, which can affect blood gas readings, were recorded. Others such as the fluid balance, urine output and total drugs administered intra-operatively were noted. Data collection was completed at 2 h post-propofol cessation and no further follow-up was required after the 2-h post-operative period.

The sample size was calculated using G*Power software version 3.1. Considering the power of 80% and the type 1 error α of 5%, the sample size required was 34 participants in each group. Considering there would likely be a 10% dropout rate for each group, an additional four patients were needed in each group. Therefore, the final sample size was 76 in total or 38 per group. All research forms were checked, compiled and entered into the IBM Statistical Package of Social Sciences (SPSS) version 26.0 and R statistical package version 3.5.0 for data analysis. For the demographic data, the gender and comorbidities are presented as frequencies and percentages, while the age and body mass index (BMI) are reported as means and standard deviations. The clinical data presented are mainly in mean values and standard deviations. The surgical duration, anaesthesia duration, volume of crystalloid used, urine output, total cumulative dose and volume of propofol used were analysed using an independent *t*-test. Further to this, repeated-measures ANOVA was used to compare the serum TG and total cholesterol via three-point testing between the two groups. Subsequently, Pearson’s chi-squared test was used to compare the two groups on the incidence of hyperTG and hypercholesterolaemia, surgical type and surgical site. The correlation between TGs and the volume and dose of propofol was explored with Pearson’s correlation analysis. Lastly, the serum-lactate and acid-base parameters for the two groups were investigated with repeated-measures ANOVA.

## Results

A total of 76 patients were randomised into the propofol 1% or 2% group but two patients were then withdrawn from the study due to massive blood loss. The data for the remaining 74 participants were analysed to compare the effects of TCIs of propofol at 1% and 2% on the patients’ lipid profiles, acid-base parameters (pH, base excess [BE], bicarbonate ion [HCO_3_]) and serum lactate. There were 37 subjects in each group.

## Demographic Data

Both groups were similar in terms of age (*P* = 0.814) and BMI (*P* = 0.067) (Table 1). The mean BMI value was normal at 24.83 kg/m^2^ (SD = 4.48). More than half of the patients had at least one type of underlying comorbidity, with hypertension the most common among the study subjects.

### Clinical Data

The indications for neurosurgical intervention were tumorous (62.2%), vascular (23%), cranial decompression (9.5%) and others (5.5%). There were more craniotomies involving supratentorial regions (81.1%) than infratentorial regions (18.9%) in both groups. The overall mean surgical duration was 246 min (SD = 115.8) and the mean anaesthetic duration was 376.76 min (SD = 147.06), with no significant difference between the two groups (*P*-values of 0.322 and 0.315, respectively) (Table 2).

The mean dose and volume of propofol infusion were 3,250.82 mg (SD = 1,637.73) and 246.18 mL (SD = 161.05), respectively. The 1% propofol group received 202.61 mg more than the 2% group, which was not statistically significant. The total volume of propofol administered to the 1% propofol group was 177.98 mL higher than for the 2% group (*P* = 0.005) and the frequency of syringe changes was also lower in the 2% than the 1% group (2 [3] versus 6 [3)]; *P* < 0.001). There were no significant differences in the incidence of hypotension, hypothermia, urine output or the volume of crystalloid administered between the two groups. No desaturation event was reported during the study. Slightly more than half of the patients required a blood transfusion, and the 1% propofol group had a higher number and percentage (*n* = 26, 60.5%) than the 2% group (*n* = 17, 39.5%; *P* = 0.034).

The baseline lipid profile was taken before operating and labelled as ‘pre-op’. With regards to TG analysis (Table 3) of the 1% propofol group, there was an increase of serum TG from ‘pre-op’ to ‘intra-op’ (1.29 mmol/L [SD = 0.47] versus 1.65 mmol/L [SD = 0.64]), which subsequently fell near to the baseline at 2 h ‘post-op’ (1.52 mmol [SD = 0.77]). Meanwhile, the 2% propofol group underwent a gradual reduction of serum TG from ‘pre-op’ to ‘intra-op’ and onto ‘post-op’ (1.29 mmol/L [SD = 0.45] versus 1.43 mmol/L [SD = 0.61] versus 1.47 mmol/L [SD = 0.72]). A serum TG of more than 1.7 mmol/L was labelled as hyperTG. The percentage of hyperTG samples increased from 18.9% (‘pre-op’) to 40.5% (‘intra-op’) among those receiving propofol at 1% and from 13.5% (‘pre-op’) to 27% (‘intra-op’) among the 2% propofol group. However, there was no statistical difference of hyperTG incidence between the two groups.

The total cholesterol for both groups decreased at the ‘intra-op’ measurement point and picked up slightly at the ‘post-op’ point (propofol 1%: 4.33 mmol/L [SD = 0.89] versus 4.43 mmol/L [SD = 0.92]; propofol 2%: 4.34 mmol/L [SD = 0.86] versus 4.52 mmol/L [SD = 0.98]), as reflected in Table 3, though the subsequent readings were still lower than the ‘pre-op’ level. There was no significant difference in the mean values of cholesterol ‘pre-op’, ‘intra-op’ and ‘post-op’ between the two groups. Also, the incidence of hypercholesterolaemia, as determined via three-point measurement between the two groups, was similar. The relationship of serum TG and total cholesterol with propofol dosing was further explored using Pearson’s correlation (*r*) but no significant correlation was found (Table 4).

**Figure 1:**
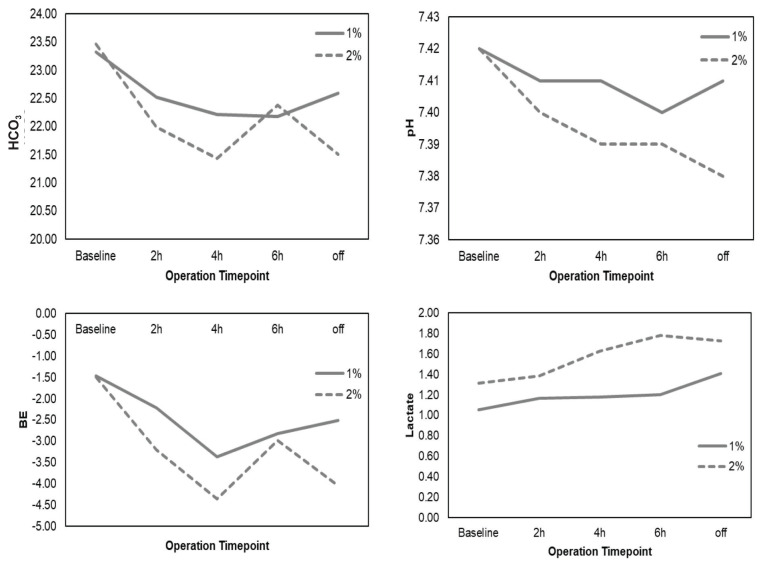
Mean acid-base balance variables values over intra-operative 2-hourly time points

The acid-base parameters taken at 2-hourly intervals intra-operatively are shown in Figure 1. The mean values were analysed using repeated-measures ANOVA. There was no significant difference in mean pH (*F*[1, 30] = 1.147; *P* = 0.293), BE (*F*[1, 30] = 0.966; *P* = 0.334), HCO_3_ (*F*[1, 30] = 0.302; *P* = 0.587) or lactate (*F*[1, 30] = 2.581; *P* = 0.119) between the two treatment groups. None of the patients experienced severe adverse events intra-operatively, such as myocardial infarction, anaphylaxis, intractable hypotension, convulsion, propofol infusion syndrome or death, while TCIs of propofol were used during the study period.

## Discussion

TCIs of propofol and remifentanil in neurosurgery have long been advocated for their favourable neurophysiological outcomes. Due to its high lipid solubility, propofol is prepared in a lipid-based emulsion. The fat emulsion contains egg phosphatide and 10% soybean oil and conventionally consists of LCTs (27). Yet, in view of the high incidence of pain on propofol injection, it is now formulated with a mixture of LCTs/MCTs at a 1:1 ratio (28). Regardless, it still poses the risk of significant lipid loading to the patient, even for short-term anaesthesia, as reported by Bhukal et al. (29).

Both concentrations of propofol used for this study are manufactured by Fresenius Kabi. The LCT/MCT ratio for propofol at 1% and 2% is similar and contains 0.1 g/mL of lipid (30–31). The lipid loading is in proportion to the volume of propofol infused. For instance, a 335.27 mL (Table 2) mean volume of 1% propofol administered during neurosurgery will translate into 41 g of lipid loading to a patient during elective neurosurgery. This value is comparable with the European Society for Parenteral and Enteral Nutrition’s (ESPEN) recommended daily dose of parenteral lipid emulsion, of 0.7 g/kg/day–1.5 g/kg/day, provided it is infused over 12 h–24 h (32).

Propofol-related hyperTG had been reported in several studies, mainly among patients with prolonged sedation in an ICU setting. Devaud et al. (33) found as many as 45% of ICU patients to have hyperTG, which was highly correlated with propofol use. Yet, data from Gottardis et al. (34) showed that lipid concentrations (serum TG and cholesterol) were not significantly influenced by propofol sedation in the ICU. These conflicting findings are possibly due to the lower dose of propofol infusion required to achieve sedation when compared to GA, along with the multitude of factors affecting critically ill patients in the ICU and potentially inducing hyperTG.

Another study pertinent to neuroanaesthesia, by Dewandre et al. (19), compared the effects of propofol at 2% and 1% in TIVA for a craniotomy and reported significantly higher plasma TGs among the 1% propofol group, though these remained within the normal range. As the study dates back to 1994, at its time of publication, remifentanil had just been introduced and TCIs models for remifentanil and propofol were not commercially available yet. The study used a combination of propofol in a BET regime and an alfentanil infusion for TIVA. There is no study at the time of writing that compares the lipid profile of TCIs of propofol at 1% and 2% in neurosurgery. All the prior studies mentioned in this article have used conventional manual TIVA. In the findings of this research, although the intra-operative propofol dosing from the 1% propofol group was slightly higher compared to the 2% group, there was no significant difference (3,351.68 mg [SD = 1,742.66] versus 3,149.07 mg [SD = 1,543.07]; *P* = 0.945) (Table 2). As expected, the cumulative propofol volume consumed was significantly higher among the 1% propofol group as the volume required was double the equivalent 2% propofol dose (335.17 mL [SD = 174.27] versus 157.19 mL [SD = 77.14]; *P* < 0.001). This translated into higher lipid loading among the patients in the 1% propofol group. The anaesthetists in the 1% propofol group changed four times more syringes (propofol 1%: 6 ***±*** 3 times versus propofol 2%: 2 ***±*** 3 times), which could have caused interruption to the surgery.

At the conclusion of TIVA, we found that the serum TG of the 1% propofol group ‘intra-op’ was elevated and higher than the 2% propofol group. There was also a greater proportion of hyperTG samples ‘intra-op’ among the 1% propofol group compared to the 2% group (40.5% versus 27%; *P* = 0.219). The ‘protective effect’ of propofol at 2% against hyperTG was not proven significant as the incidences of hyperTG and hypercholesterolaemia for the two groups were similar (Table 3) and the mean values of serum TG for both groups remained within the normal limits. This means it is likely that the recruited participants in this study, who were free from metabolic syndromes, familial hyperTG and liver disease, could instigate sufficient TG clearance to counteract excessive lipid loading from propofol. Further studies are needed with higher cumulative doses and volumes of propofol or prolonged propofol infusion, to determine whether there are any significant effects of using 2% versus 1% propofol.

Inevitably, *n* = 21 (56.8%) and *n* = 22 (59.5%) of our recruited patients from the 1% and 2% propofol groups, respectively, continued propofol infusions post-operatively as they were deemed unsuitable for fast-track extubation. Possible causes of the delayed extubation were cerebral aneurysm, tumour size, prolonged surgery, unfavourable Glasgow coma score (GCS) and the presence of hydrocephalus. In the majority of these cases, the patients were switched to propofol infusions post-operatively at 1 mg/kg/h–2 mg/kg/h in the high-dependency unit. The TG measured upon cessation of TCIs of propofol and at two hours post-operatively did not show significant elevation and thus did not reflect the intra-operative lipid load.

Though we did not establish a difference in the incidence of hyperTG among the 1% and 2% propofol groups (Table 2), the choice of propofol concentration should be made with caution among susceptible patients such as those with familial hyperTG or a metabolic syndrome. Extrapolation of the hyperTG risk of propofol usage in intensive care should be done with care. In cases where ICU patients receive prolonged infusions of propofol, lipid profile monitoring is warranted as well as being alert for PRIS.

A high dose of propofol can inhibit mitochondrial respiration, which can cause metabolic acidosis (35). In phase-two propofol metabolism, glucuronide and sulphate metabolites are produced. The intermediate dehydroxylated products can potentially be converted to quinones, which are toxic (36–37). Alternatively, metabolic acidosis can be related to lipid loading separate from the action of propofol (31, 35–36). However, limited studies have been carried out to evaluate the incidence of metabolic acidosis linked with propofol use. Notably, there are case reports that illustrate the sole manifestation of non-fatal PRIS as reversible metabolic acidosis (37–39).

Various factors can influence the acid-base status and serum lactate. Possible intra-operative causes of metabolic acidosis were evaluated in this research. Other than significantly more 1% propofol participants receiving a blood transfusion (70.3% versus 45.9%; *P* = 0.034), there were no significant differences in the urine output, incidence of hypotension or hypothermia that may have affected the acid-base parameters or serum lactate. Nevertheless, our study showed no significant differences in the mean pH, BE, HCO_3_ and lactate between the two groups. Clinical metabolic acidosis should, therefore, not affect the choice of propofol concentration to use.

There are several limitations to this study that must be acknowledged. First, we excluded patients such as those with familial hyperTG, liver disease and metabolic syndrome as their TG clearance was deemed to be affected. Hence, these data may not be suitable to extrapolate to the general population, which includes a cluster at risk of developing hyperTG. Second, as there was no reported kidney disease as a comorbidity in any of the patients in either group, we did not review the baseline or post-operative renal profile, which could have reflected the efficacy of acid elimination. Further to this, the haematocrit level and serum creatinine kinase level were not included among the variables in this study, though we recognise that these can be confounders of metabolic acidosis and acute kidney injury, respectively. Finally, this was a single-centre interventional study with a small sample size. A more robust study with a larger sample size is needed to further investigate the TG difference afforded by propofol at 2%.

## Conclusion

Propofol at 1% and 2% causes a similar incidence of hyperTG during neuroanaesthesia. However, we should tailor the propofol concentration in TIVA for susceptible patients. Neither propofol at 1% nor 2% affect the acid-base balance during TIVA. The benefits of propofol at 2% were the lower volume used and fewer syringe changes, which could minimise anaesthesia interruption throughout surgery.

## Figures and Tables

**Table 1:** Demographic data

Variables	All	Group		*P*-value

		Propofol 1% (*n* = 37)	Propofol 2% (*n* = 37)	
Age (years old)	43.72 (14.19)	43.32 (13.40)	44.11 (15.11)	0.814 a
Gender				0.418^b^
Male	34 (45.9%)	19 (51.4%)	15 (40.5%)	
Female	40 (54.1%)	18 (48.6%)	22 (59.5%)	
Comorbid				0.262^b^
Diabetes mellitus	13 (17.6%)	6 (16.2%)	7 (18.9%)	
Hypertension	26 (35.1%)	14 (37.8%)	12 (32.4%)	
Dyslipidemia	2 (2.7%)	2 (5.4%)	0 (0.0%)	
Others	20 (27.0%)	5 (13.5%)	15 (40.5%)	
NIL	31 (41.9%)	18 (48.6%)	13 (35.1%)	
BMI (kg/m^2^)	24.83 (4.48)	23.87 (3.34)	25.78 (4.48)	0.067 a

Notes: Data is presented as frequency (percentage) or mean (SD);

a Independent *t*-test;

b Pearson’s chi-squared test;

A *P*-value less than 0.05 indicate a statistically significant difference

**Table 2:** Description of surgical and anaesthetic characteristic variables in propofol 1% and 2% groups

Variables	All	Group		*P*-value

		Propofol 1% (*n* = 37)	Propofol 2% (*n* = 37)	
Surgical type				0.280 b
Decompression	7 (9.5%)	3 (8.1%)	4 (10.8%)	
Tumour	46 (62.2%)	27 (73%)	19 (51.4%)	
Vascular	17 (23%)	7 (18.9%)	10 (27%)	
Others: empyema drainage	1 (1.4%)	0 (0.0%)	1 (2.7%)	
Others: epilepsy	3 (4.1%)	0 (0.0%)	3 (8.1%)	
Surgical site				0.433 b
Infratentorial	14 (18.9%)	8 (21.6%)	6 (16.2%)	
Supratentorial	60 (81.1%)	29 (78.4%)	31 (83.8%)	
Surgical duration (min)	246.00 (115.80)	286.51 (126.24)	205.47 (88.78)	0.212 a
Anaesthesia duration (min)	376.76 (147.06)	420.46 (153.92)	333.05 (127.42)	0.285 a
Sterofundin volume (mL)	2,250 (1062.98)	2,481.08 (1142.33)	2,018.92 (936.26)	0.439 a
Normal saline volume (mL)	774.39 (552.62)	770.41 (517.19)	778.38 (593.08)	0.478 a
Dose of propofol (mg)	3,250.82 (1,637.73)	3,351.68 (1,742.66)	3,149.07 (1,543.07)	0.895 a
Volume of propofol (mL)	246.18 (161.05)	335.17 (174.27)	157.19 (77.14)	0.015 a
Number of syringe change		6 ± 3	2 ± 3	
Blood transfusion				0.124 b
Yes	43 (58.1%)	26 (70.3%)	17 (45.9%)	
No	31 (41.9%)	11 (29.7%)	20 (54.1%)	
Hypotension				0.789 b
Yes	27 (36.5%)	14 (37.8%)	13 (35.1%)	
No	47 (63.5%)	23 (62.2%)	24 (64.9%)	
Desaturation				
< 90%	0 (0.0%)	0 (0.0%)	0 (0.0%)	–
> 90%	74 (100.0%)	37 (100.0%)	37 (100.0%)	
Hypothermia				0.055 b
< 35 °C	14 (18.9%)	10 (27.0%)	4 (10.8%)	
> 35 °C	60 (81.1%)	27 (73.0%)	33 (89.2%)	
Urine output (mL/kg/h)	3.56 (2.21)	3.69 (2.43)	3.43 (1.98)	0.218 b

Notes: Data is presented as frequency (percentage) or mean (SD);

a Independent *t*-test;

b Fisher’s exact test;

*P*-value less than 0.05 indicate a statistically significant difference

**Table 3:** Comparison of serum triglyceride and total cholesterol characteristics between propofol 1% and 2% groups

Variables	Group		*P*-value

	Propofol 1% (*n* = 37)	Propofol 2% (*n* = 37)	
Triglyceride (mmol/L)			
Pre-op	1.29 (0.47)	1.29 (0.45)	0.140 a
Intra-op	1.65 (0.64)	1.43 (0.61)	
Post-op	1.52 (0.77)	1.47 (0.72)	
No. of patient with hyperTG^1^			
Pre-op	7 (18.9%)	5 (13.5%)	0.528 b
Intra-op	15 (40.5%)	10 (27%)	0.219 b
Post-op	12 (32.4%)	13 (35.1%)	0.806 b
Cholesterol (mmol/L)			
Pre-op	4.95 (1.01)	4.77 (1.02)	0.253 a
Intra-op	4.33 (0.89)	4.34 (0.86)	
Post-op	4.43 (0.92)	4.52 (0.98)	
No. of patient with hypercholesterolemia^2^			
Pre-op	4 (10.8%)	2 (5.4%)	0.394 b
Intra-op	1 (2.7%)	0 (0.0%)	0.314 b
Post-op	1 (2.7%)	1 (2.7%)	1.000 b

Notes: Data is presented as frequency (percentage) or mean (SD);

a repeated-measures ANOVA test (between group comparison);

b Pearson’s chi-squared test;

*P*-value less than 0.05 indicate a statistically significant difference;

1 Number of patients with hyperTG > 1.7 mmol/L in frequency (percentage);

2 Number of patients with hypercholeseterolemia > 6.3 mmol/L in frequency (percentage)

**Table 4:** Pearson’s correlations coefficient (*r*) between lipid profile with cumulative dose and volume of propofol infused among propofol 1% and 2% (*N* = 74) groups

Variables	Propofol 1% (*n* = 37)		Propofol 2% (*n* = 37)	

	Dose *r* (*P*-value)	Volume *r* (*P*-value)	Dose *r* (*P*-value)	Volume *r* (*P*-value)
Triglyceride				
Intra-op	0.242 (0.149)	0.242 (0.149)	−0.094 (0.579)	−0.097 (0.568)
Post-op	0.220 (0.190)	0.220 (0.190)	0.053 (0.756)	0.051 (0.766)
Cholesterol				
Intra-op	−0.039 (0.818)	−0.039 (0.818)	−0.078 (0.647)	−0.077 (0.650)
Post-op	−0.050 (0.769)	−0.050 (0.769)	0.001 (0.995)	0.002 (0.992)

Note: Data is presented as Pearson’s correlation coefficient (*r*); a correlation is significant at *P*-value < 0.05 (2-tailed)
